# The evolution of a rare mammalian trait – benefits and costs of male philopatry in proboscis bats

**DOI:** 10.1038/s41598-017-15990-6

**Published:** 2017-11-15

**Authors:** Linus Günther, Mirjam Knörnschild, Martina Nagy, Frieder Mayer

**Affiliations:** 1 Museum für Naturkunde, Leibniz Institute for Research on Evolution and Biodiversity, Invalidenstrasse 43, D–10115 Berlin, Germany; 2 Free University Berlin, Institute of Biology, Animal Behavior Lab, Takustrasse 6, D–14195 Berlin, Germany; 3 Smithsonian Tropical Research Institute, Roosevelt Avenida, Tupper Building 401, Balboa, Panama

## Abstract

While inbreeding avoidance is widely accepted as the major driver of female natal dispersal, the evolution of male philopatry is still poorly understood and discussed to be driven by male mating strategy, mate competition among male kin and kin cooperation. During a twelve-year study, we gathered detailed genetic and observational data of individually marked proboscis bats to assess the degree of male philopatry as well as its costs and benefits to improve the understanding of its evolution. Our results reveal several patrilines with simultaneous presence of closely related males and a small proportion of unrelated immigrant males in their colonies. Philopatric males benefit from avoiding the costs of immigration into foreign colonies through significantly longer tenure, better integration (i.e. frequent nocturnal presence in the colonies) and consequently significantly higher reproductive success compared to immigrant males. Finally, we illustrate that despite a high proportion of philopatric males in the groups, the number of closely related competing males is low. Thus, the hypothesised costs of mate competition among male kin seem to be low in promiscuous mammalian societies with unrelated females and a small degree of male immigration and are readily outweighed by the benefits of staying in the natal group.

## Introduction

The decision to leave or stay and reproduce in the natal group has fundamental effects on an individual’s life as well as the genetic and social structure of societies and the demography of species^1^. Thus, as one of the most important life history traits, natal dispersal patterns have been of great interest for over 40 years^1–4^. Dispersal patterns are usually sex-biased in birds and mammals with one sex being faithful to the natal group or area (philopatry), while individuals of the other sex are prone to disperse prior to sexual maturity (natal dispersal). Male-biased dispersal (MBD) is the general pattern in mammals, whereas female-biased dispersal (FBD) prevails in the majority of bird species^3,5,6^. Female natal dispersal in mammals builds an exception to this rule and is consistently associated with male breeding tenures that commonly exceed the age of females’ first conception^7,8^. This suggests that inbreeding avoidance is the main driver of female natal dispersal in mammals^7,8^. In contrast, exceptional male philopatry in mammals is less well understood and raises the question how this rare mammalian life history trait could evolve.

Several benefits and costs of staying in the natal group that affect reproductive success of philopatric males need to be considered as potential drivers in the evolution of male philopatry. First, empirical examples suggest that philopatric males may benefit from cooperating with their relatives (e.g. reviewed for nonhuman primates in^9^, red grouse, *Lagopus lagopus scoticus* ^10^), thus kin cooperation is discussed as an important driver in the evolution of male philopatry (e.g.^11,12^). However, it is not clear whether kin cooperation is an ultimate factor in the evolution of male philopatry or if male philopatry is rather a precondition of kin cooperation among males (e.g.^9^). Second, philopatric individuals may simply benefit from avoiding high costs associated with dispersal (e.g.^13–17^). While mortality risk might remain unchanged or increase during dispersal (e.g. white-footed mouse *Peromyscus leucopus* ^18^, root vole *Microtus oeconomus* ^19^), dispersing individuals are likely to lack familiarity with the distribution of resources, which in turn may decrease feeding efficiency (e.g. African elephant *Loxodonta africana* ^20^, meerkat *Suricata suricatta* ^21^). Moreover, immigrant individuals are often likely to be attacked by resident group members^22–25^ and worse physical conditions (i.e. less weight or smaller skeletal traits) of dispersers compared to philopatrics^13,26–28^ can lead to competitive disadvantages^27–29^. As a result philopatric individuals may have higher reproductive potential^15,30^. However, staying in the natal group can also be associated with costs. Philopatry can lead to mating with close kin and inbreeding depression has repeatedly been shown to have severe fitness costs (e.g.^31,32^). Moreover, philopatric males in polygynous systems may incur costs from competing for mates with close kin (i.e. local mate competition – LMC). Since LMC lowers a male’s inclusive fitness^33,34^, its avoidance is assumed to be one of the important selective pressures causing male natal dispersal in mammals^5,11,35^. However, there are examples of mammalian groups with multiple reproducing male kin (e.g., lions *Panthera leo* ^36^, bottlenose dolphins *Tursiops sp*.^37^, greater sac-winged bat *Saccopteryx bilineata* ^38^). This raises the question which benefits allow multiple related males to reproduce in the same social group, and how severe the potential costs of local mate competition are that might need to be overcome. Due to the difficulties in assessing fitness differences between philopatric and dispersing males, only a few mammalian species have been studied regarding differences in reproductive success of philopatric and dispersing males (reviewed in^39,40^). The few examples of mammalian social groups consisting of philopatric and immigrant males – as e.g. suggested for the Neotropical proboscis bat^41^ – are a rare opportunity to discover proximate effects that could lead to potential differences in fitness and test hypotheses on the evolution of male philopatry.

In our focal species, the proboscis bat *Rhynchonycteris naso* it was shown that at least half of the male colony offspring settles in the natal colony, where some of them reproduce, while all females disperse from their natal colony prior to reproduction^41^. In addition, colony offspring are fathered by resident males and males have a long reproductive tenure^41,42^, suggesting that fathers roost together with their sons and other close kin in a colony. This would mean that males compete with close kin for access to territories and female mating partners and that male *R. naso* incur LMC costs. As females disperse prior to reproduction, inbreeding does not appear to be an issue for male *R. naso*. Interestingly, Nagy *et al*.^41^ also reported some incidences of male immigration, but were unable to quantify the degree of male philopatry and immigration and thus, the consequences on the genetic structure of *R. naso* groups and fitness differences between philopatric and immigrant males remain unknown.

*Rhynchonycteris naso* roosts in social groups that are stable year–round, consist of up to 50 individuals with males and females at about equal numbers and occupy exposed parts of tree trunks, branches, vines or man–made structures^43^. Day-roosts are also occupied at night when bats return from foraging bouts. They reproduce within one seasonal mating period (SMP; October/November) and one postpartum oestrus mating period (PEMP; April/May)^41,42,44^. Male proboscis bats exhibit a territory based mating strategy with aspects of both, resource-defence and direct female-defence^42^. At night, territorial males constantly occupy a territory that might or might not include females, while non-territorial males are either absent or float between different sites in the roost. By contrast, due to assumingly high predation risk in the exposed roosting sites, all male and female group members roost in cohesive groups during the day in the territory of one male and relocate together to various different territories of a roost – probably to avoid unfavourable changes in roost site temperature (MN unpublished data). This starts with several individuals simultaneously changing roost site followed by all other group members within seconds to minutes. While roosting together as one group during the day, territorial males must defend females directly against all other male competitors roosting within their territory. Thus, within the same social group a territorial male is dominant at one site (in his territory), but non-dominant once the whole group relocates to another male’s territory. This mating system and male philopatry leads to simultaneous reproduction by multiple males. Details on the relatedness of these competing males are currently lacking, thus the potential for LMC in *R. naso* remains unknown.

Here, we combine long–term behavioural observations of individually marked proboscis bats with genetic parentage analyses to assess the degree of male philopatry and immigration and the consequences for male relatedness structure of three free ranging *R. naso* colonies. Further, we explore possible benefits and costs of philopatric and dispersing males by comparing their reproductive success. Specifically, we hypothesise that (1) the majority of present adult males in *R. naso* colonies is philopatric, (2) philopatric males have advantages compared to dispersing males that result in higher fitness, and (3) a high proportion of male philopatry leads to the presence of closely related males in *R. naso* groups and thus to LMC.

## Results

### Male dispersal, philopatry and immigration and the impact on relatedness structures

**Figure 1:**
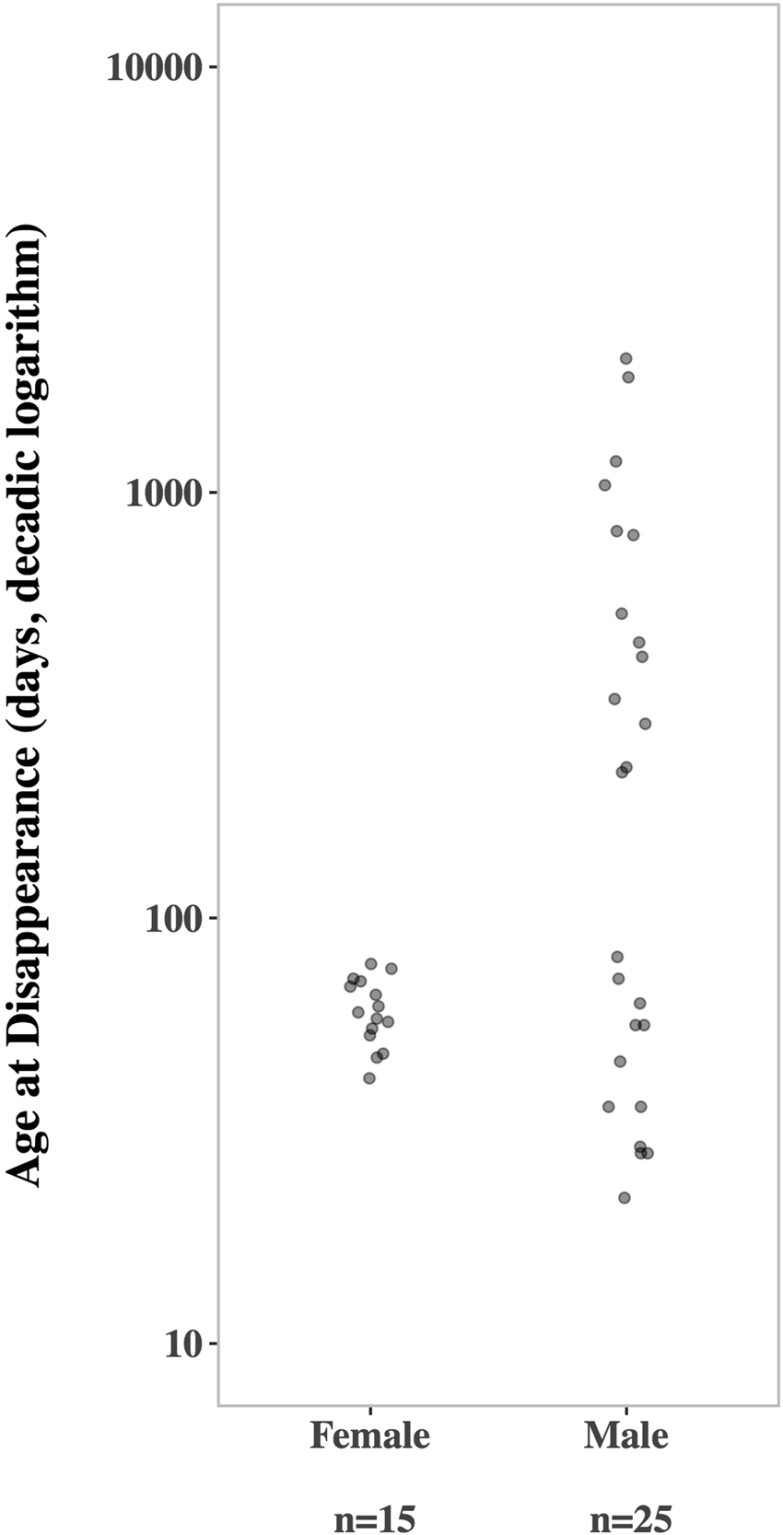
The age of disappearance from the natal colony C5 of female and male colony offspring. All female offspring dispersed prior to reproduction. Individuals with information on birth date and disappearance time born between 2008 and 2014 are shown. The observation of these individuals ended in November 2016. Please note the logarithmic scale of the y-axis.

In accordance with Nagy *et al*.^41^, in our focus colony C5 both sexes differed in the duration from birth until disappearance (i.e. death or dispersal) from the natal colony. All females disappeared very early from their natal colonies at a median age of 58.0 days (*n* = 15; range: 42–78). Some males disappeared from their natal colonies at a similar age as the females (*n* = 12, median: 41.0 days, range: 22–81). The fact that almost half of all male immigrants (*n* = 19) were juvenile (*n* = 5) or subadult (*n* = 3) indicates that at least parts of these young disappearing males dispersed to other colonies. However, the majority of males stayed longer and became colony residents; these males are regarded as philopatric (*n* = 13, median: 519 days, range: 220–2064; see Fig. 1).

In our three study colonies Cabina 5 (C5), Riverstation (RS) and Casa Grande (CG), on average 83.3% of adult males with information on dispersal status that were present after the start of 2011 were born in their colonies of residence and were, thus, philopatric (C5: 75.0%, *n* = 28; RS: 75.0%, *n* = 12; CG: 100.0%, *n* = 15). While 25.0% in C5 and RS were immigrant males, no immigration was detected in CG (see Table 1). We decided to use the year 2011 as a starting point for the analyses since the sampling/banding rate was comparatively high beginning with 2011 (mean: 87.7%, see supplementary Figure S1). However, due to incomplete sampling also after 2011, there was a proportion of adult males with no information on dispersal status (C5: 17.9% *n* = 33; RS: 36.8%, *n* = 19; CG: 21.1%, *n* = 19).

**Table 1:** Number of adult males with information on dispersal status that were present in the three study colonies between 2011 and 2015 (Cabina 5) or 2011 and 2014 (Riverstation, Casa Grande), respectively.

Colony	Period	Adult males	Philopatric	Immigrant
Cabina 5	2011–2015	28	75.0%	25.0%
Riverstation	2011–2014	12	75.0%	25.0%
Casa Grande	2011–2014	15	100.0%	0.0%
		Mean	83.3%	16.7%

The proportion of philopatric and immigrant males is given.

**Figure 2:**
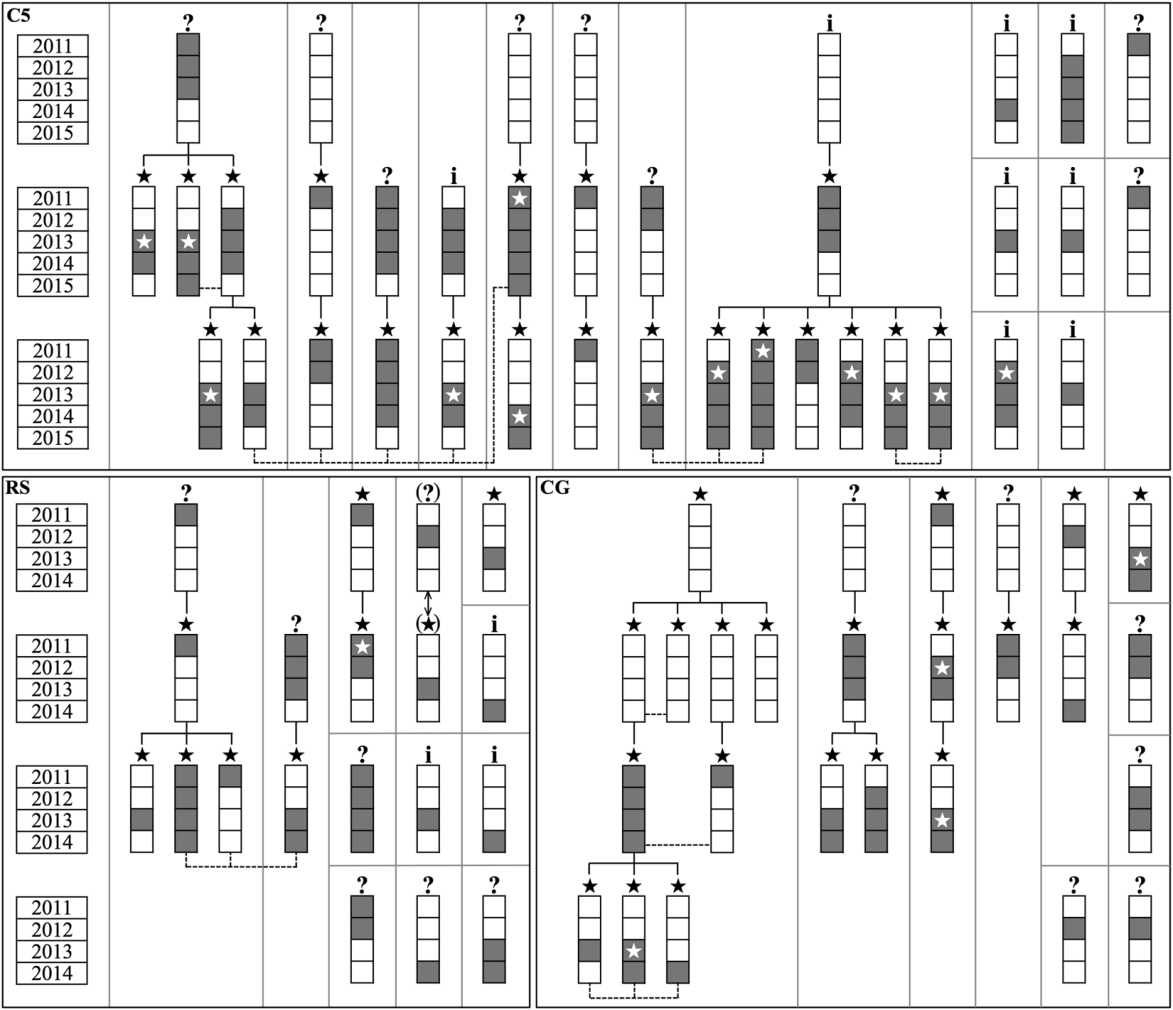
Patrilines of the three study colonies Cabina 5 (C5), Riverstation (RS) and Casa Grande (CG). All present adult males and – if assigned – their male ancestors present prior to 2011 are shown. Each column represents an individual male. Grey columns indicate the male’s presence. Solid lines indicate a father-son relationship and dashed lines beneath columns indicate males sharing the same mother. Philopatric males are marked with ‘★’, immigrants with ‘**i**’ and males with unknown dispersal status with ‘**?**’. If a male was caught as juvenile between 2011 and 2015, the year of birth is indicated with a white star in the respective columns. Please note that two philopatric adult males transferred permanently from C5 to RS, thus are listed in both colonies. The direction of one father-son relationship in RS could not be solved (marked with a two-sided arrow).

Adult males that were present after 2011 and their assigned ancestors (C5: *n* = 37, RS: *n* = 19, CG: *n* = 26) formed a maximum of 16 (C5), twelve (RS) and ten (CG) patrilines each consisting of 1–10 males (see Fig. 2). The true number of patrilines may be lower since some of them are likely to have coalesced prior to our study period. In C5, 78.4% of the males belonged to eight patrilines with at least two members (range: 2–8 members) while the remaining males belonged to eight patrilines, each with one member. In RS, 57.9% of the males belonged to four patrilines with 2–5 members and the remaining males to eight patrilines with one member. In CG, five patrilines with 2–10 members comprised 82.1% of all males while the remaining males belonged to five one-male patrilines. In all three colonies, on average 72.3% of the males belonged to patrilines with at least two members (i.e. father and son; Fig. 2). In total, the parentage analyses (CERVUS) and patriline reconstructions resulted in 52 male pairs with the relatedness class of *r* = 0.5 (C5 *n* = 24, RS *n* = 8, CG *n* = 20), 63 male pairs with the relatedness of *r* = 0.25 (C5 *n* = 40, RS *n* = 7, CG *n* = 16) and 10 male pairs with the relatedness class of *r* = 0.125 (C5 *n* = 2, RS *n* = 0, CG *n* = 8). The total number of possible male pairs was *n* = 666 in C5, *n* = 171 in RS and *n* = 325 in CG. Further, the mean calculated (KINGROUP) pairwise relatedness among adult males present during each of five (RS, CG) and six (C5) mating periods between 2011 and 2015 was low (see Table 2). This suggests that the majority of male pairs in a social group/colony was distantly related or unrelated.

**Table 2:** Mean pairwise relatedness among adult males present (*n*) during mating periods [‘postpartum oestrous mating period’ (PEMP) and ‘seasonal mating period’ (SMP)] in the three study colonies.

Colony	PEMP 2011		PEMP 2013		SMP 2013		PEMP 2014		SMP 2014		SMP 2015	
	*n*	Mean r ( + /−SD)	*n*	Mean r ( + /−SD)	*n*	Mean r ( + /−SD)	*n*	Mean r ( + /−SD)	*n*	Mean r ( + /−SD)	*n*	Mean r ( + /−SD)
Cabina 5	11	0.06 (0.20)	13	0.03 (0.19)	17	0.01 (0.17)	15	0.001 (0.15)	14	0.04 (0.15)	10	0.02 (0.15)
River-station	7	0.06 (0.22)	9	0.05 (0.15)	5	0.02 (0.22)	5	−0.01 (0.15)	4	−0.02 (0.07)	n.a.	n.a.
Casa Grande	5	0.08 (0.15)	5	0.09 (0.17)	7	0.13 (0.26)	6	0.03 (0.15)	5	0.11 (0.26)	n.a.	n.a.

Mean relatedness and standard deviation was calculated for well-sampled mating period with census observations.

### Advantages of philopatry vs. dispersal

#### Minimum male tenure

**Figure 3:**
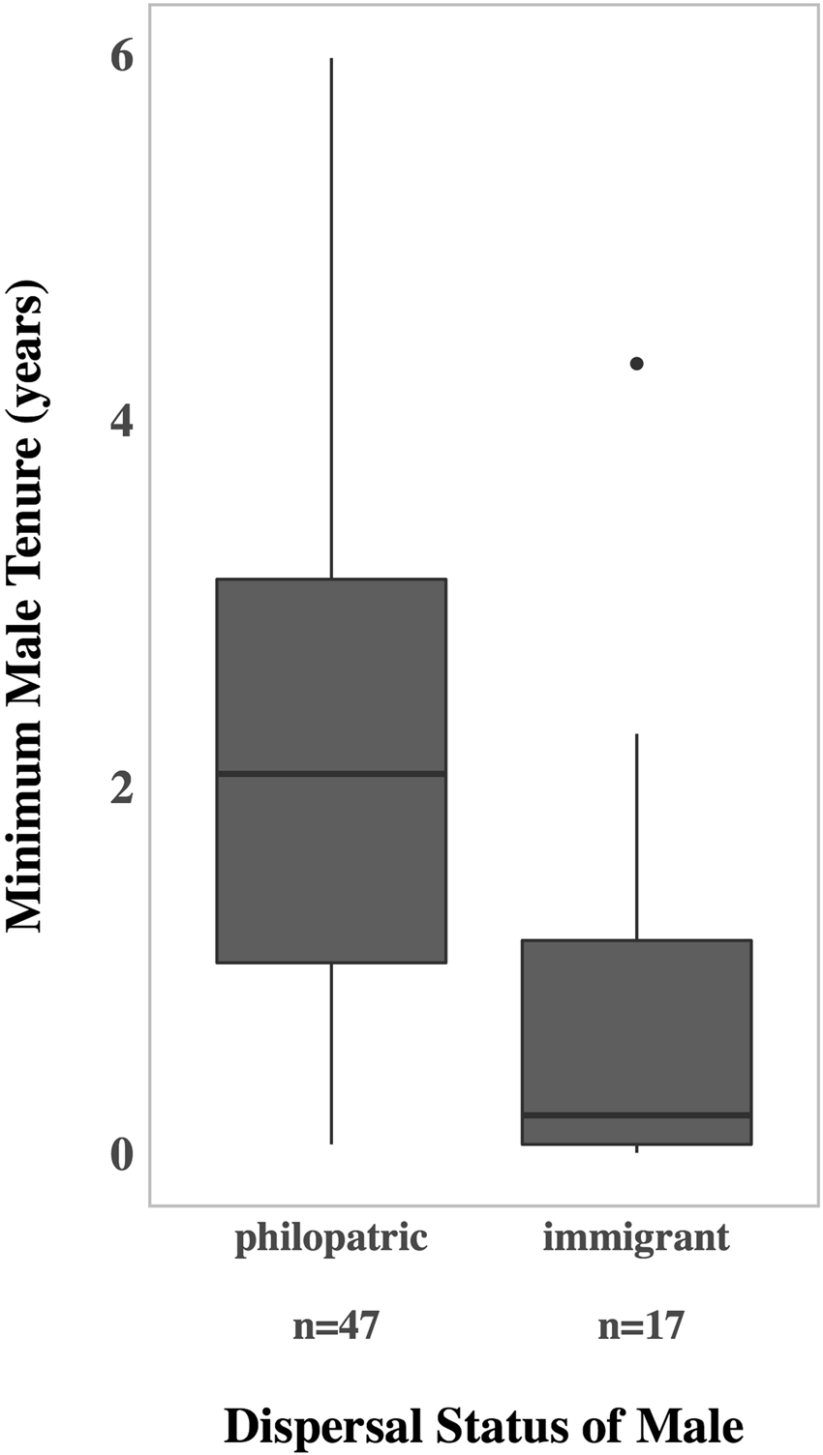
Minimum individual tenure of philopatric and immigrant males. Males caught between 2005 and 2014 are included. The observation of these individuals ended in October 2015 (RS), July 2016 (CG) and November 2016 (C5). The difference is statistically significant with *p* < 0.001. Please note that two philopatric adult males transferred permanently from C5 to RS, thus are included in the calculations with their tenure as philopatric and with their tenure as immigrant males.

To assess the success of males regarding long-term integration into a colony, we calculated the minimum tenure for philopatric males and immigrant males across all study colonies. Philopatric males had a significantly longer minimum tenure (median: 2.07 years, range: 0.05–5.98, *n* = 47) compared to immigrant males (median: 0.21 years, range: 0.003–4.31, *n* = 17; Mann–Whitney *U*-test: *n*_philopatrics_ = 47, *n*_immigrants_ = 17, *U* = 657.5, *p* < 0.001, see Fig. 3). Forty-three (91.5%) of the philopatric males disappeared (i.e. died or dispersed as adult) before the end of the study, but only two of these were observed to immigrate into another study colony at an age of 286 and 327 days, respectively. Due to the lack of information on the start (*n* = 21 philopatric males, *n* = 2 immigrant males) and the end (*n* = 4 philopatrics, *n* = 1 immigrant) of several males’ presence, the actual average tenures exceed our minimal estimates.

#### Nocturnal presence and partitioning in mating related behaviour

During four mating periods with detailed observational data in our focus colony C5 (2013 and 2014), 23 adult males were present during the day (*n* = 16 philopatrics, *n* = 7 immigrants). While all philopatric males were observed to be present during on average 53.4% nights (range: 11.4–93.8%), only three of the seven immigrant males were observed in the roost at night. These three immigrant males showed a similar average individual nocturnal presence of 61.9% (range: 38.3–75.9%) as philopatric males.

Eight (50.0%) philopatric males performed 71.9% of all copulations (*n* = 147) while three (42.9%) of the immigrant males (two of these with nocturnal presence) performed 21.2% of the observed copulations. Two males with unknown dispersal status performed the remaining 6.8% of all observed copulations. Further, all philopatric males were involved in 77.6% of all observed copulation attempts (*n* = 785), while four (two of these with nocturnal presence) of the seven immigrants performed 16.0% of all copulation attempts. The remaining 6.4% were performed by two males with unknown dispersal status.

#### Territoriality

In the focus colony C5 median minimum tenure until becoming territorial was 1.73 years (*n* = 4 males, range: 1.46–2.19). For three of these males we have the exact age, while one of the males was caught as adult. Thus, actual time span until becoming territorial exceed our minimal tenure estimates. Median minimum tenure as territorial male was 1.95 years (*n* = 8 males, range: 0.59–3.38). As four males were already territorial in 2013 and two males were still territorial at the end in 2016 the actual median tenure of territorial males exceeds our minimal tenure estimates. Nine (37.5%) of the 24 philopatric males were or became territorial, while only two (14.3%) of the 14 immigrant males became territorial. However, the probability of becoming a territorial male was not statistically different between philopatric and immigrant males (Pearson’s chi-squared test: chi-squared = 2.66, df = 1, p-value = 0.10).

#### Physical differences and competitive advantages for philopatric males

**Figure 4:**
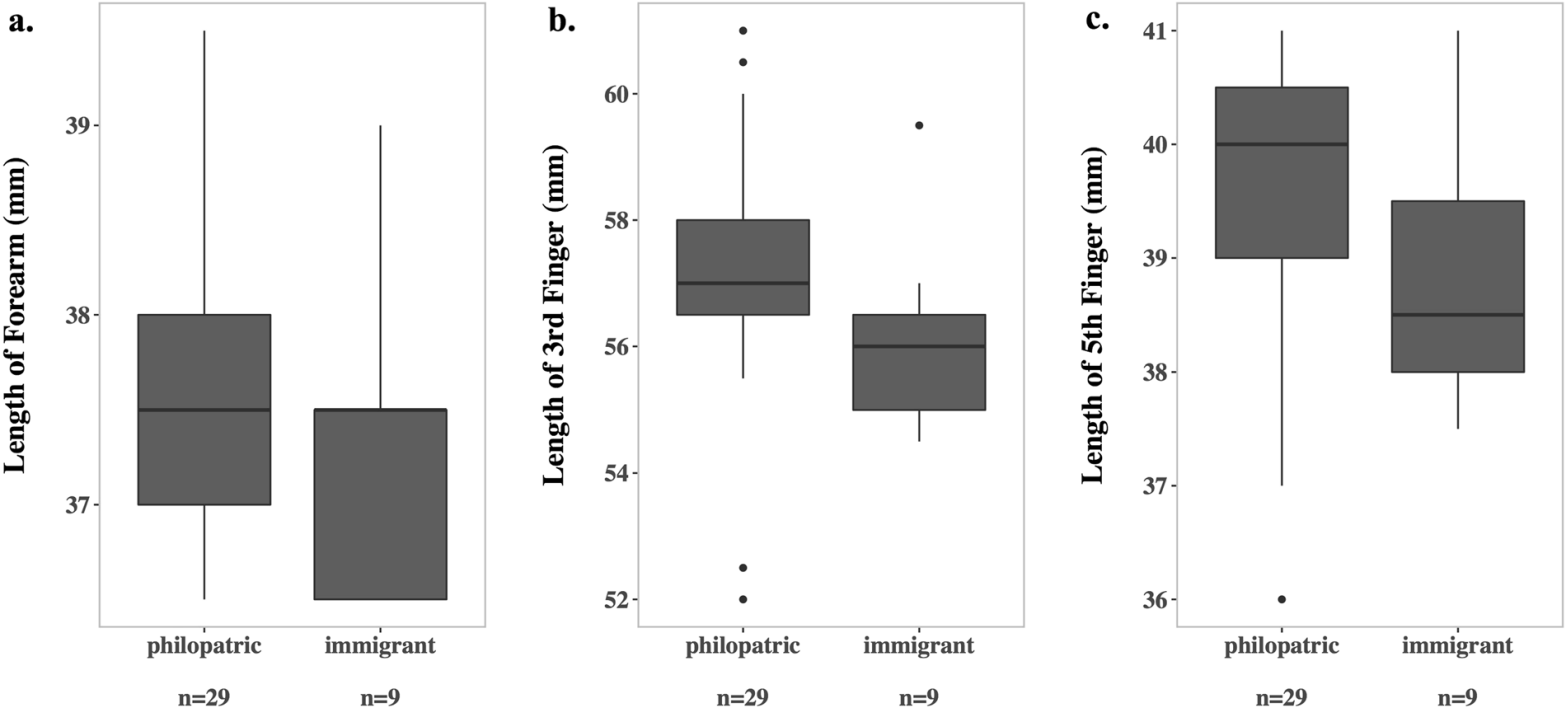
Size comparison of philopatric and immigrant males. Length of forearm, third and fifth finger of adult philopatrics (C5: *n* = 15, RS: *n* = 3, CG: *n* = 10) and adult immigrants (C5: *n* = 7, RS: *n* = 2) is shown. All measurements were performed by the same person (L.G) at a minimum age of one year, when all bats were fully-grown. All differences are statistically significant with p < 0.001.

Physical aggression is likely to be involved in the immigration process and is frequent among resident males. In this context, large body size might convey competitive advantages during immigration and male-male interactions. Thus, we tested for size differences between adult philopatric and immigrant males across all study colonies. Forearm, third and fifth finger of adult philopatric males were significantly longer compared to adult immigrant males (Mann–Whitney *U*-tests: *n*_philopatrics_ = 29, *n*_immigrants_ = 9: Forearm: *U* = 435, *p* < 0.001; Third finger: *U* = 435, *p* < 0.001; Fifth finger: *U* = 435, *p* < 0.001; see Fig. 4). Due to dispersal of some males at an early age, we only used post-dispersal males that immigrated into our study colonies and were measured as fully-grown adults (minimum age of one year). The same was done with philopatric males.

#### Male reproductive success

**Figure 5:**
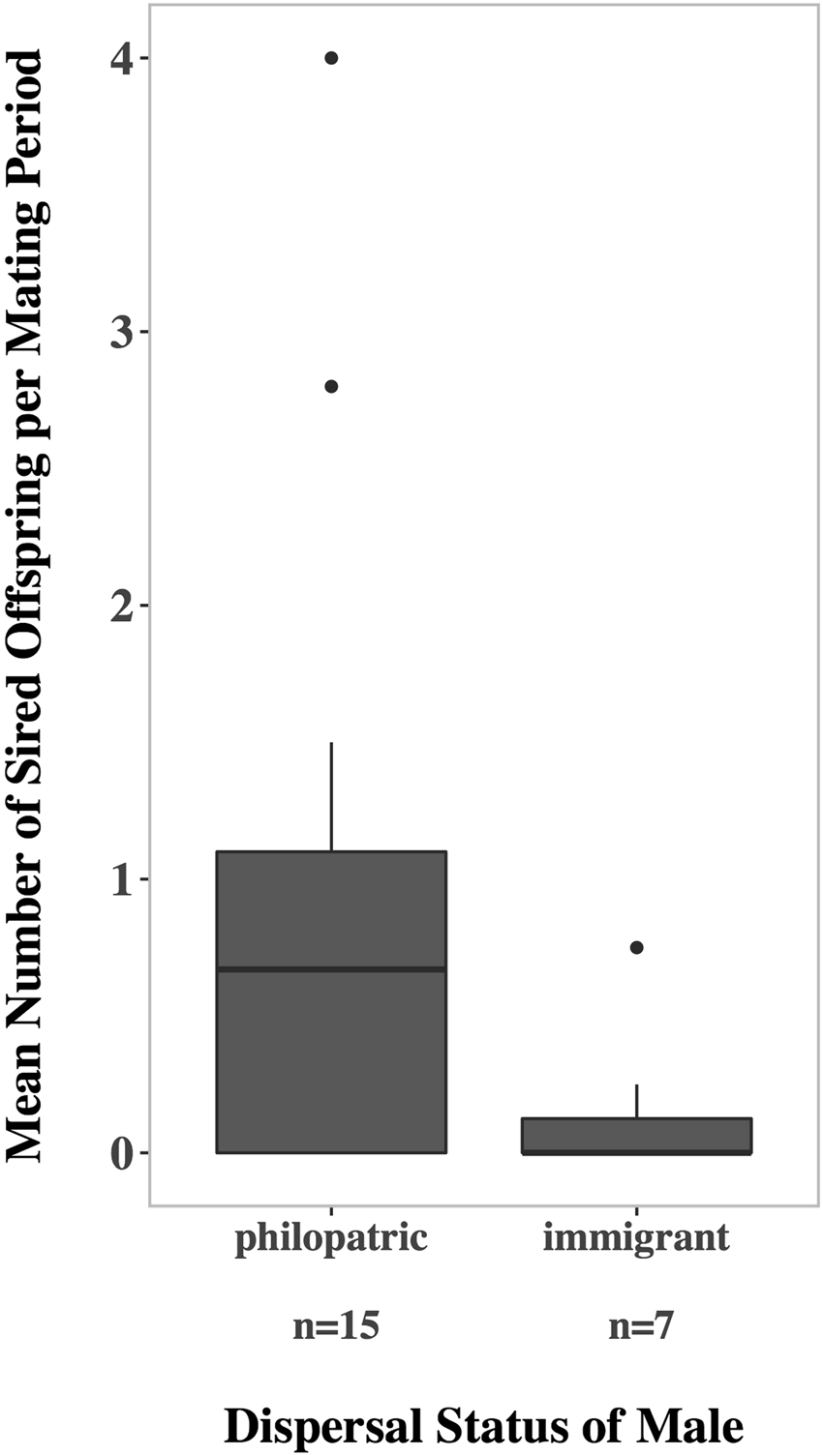
Mean number of sired offspring per mating period by philopatric and immigrant males. Offspring of fathers with known dispersal status from six different mating periods between 2010 and 2014 are included (*n* = 45 offspring). The number of sired offspring by each male is averaged for the six mating periods the males were present as adult individuals. The difference is statistically significant with *p* = 0.029.

We were able to determine paternity for all 57 colony offspring sired during six mating periods between 2010 and 2014 in our focus colony C5. Colony offspring were either observed to be nursed in the colony and genetically confirmed (*n* = 49) or the mother was genetically assigned and present in the colony and season the pup was sampled in (*n* = 8). Of the colony offspring that were sired by males with known dispersal status (*n* = 45), 91.1% were sired by ten of 15 adult philopatric males, while only 8.9% were sired by four of seven adult immigrant males. Philopatric and immigrant males were present during at least one of the six sampled mating periods (15 philopatric males on average: 2.5 mating periods, range: 1–5; 7 immigrant males on average: 2.4 mating periods, range: 1–4). Six males without information on dispersal status and one member of another colony (RS) sired the remaining twelve colony offspring. Mean individual reproductive success per mating period was significantly higher for philopatric than immigrant males (Fig. 5; Median reproductive success: philopatric males = 0.67, IQR = 0.00–1.10; immigrant males = 0.00, IQR = 0.00–0.13; Mann-Whitney *U*-test: *n*_philopatrics_ = 15, *n*_immigrants_ = 7, *U* = 78.5, p = 0.029).

### Disadvantages of philopatry

#### Relatedness of present adult males and mate competition

**Figure 6:**
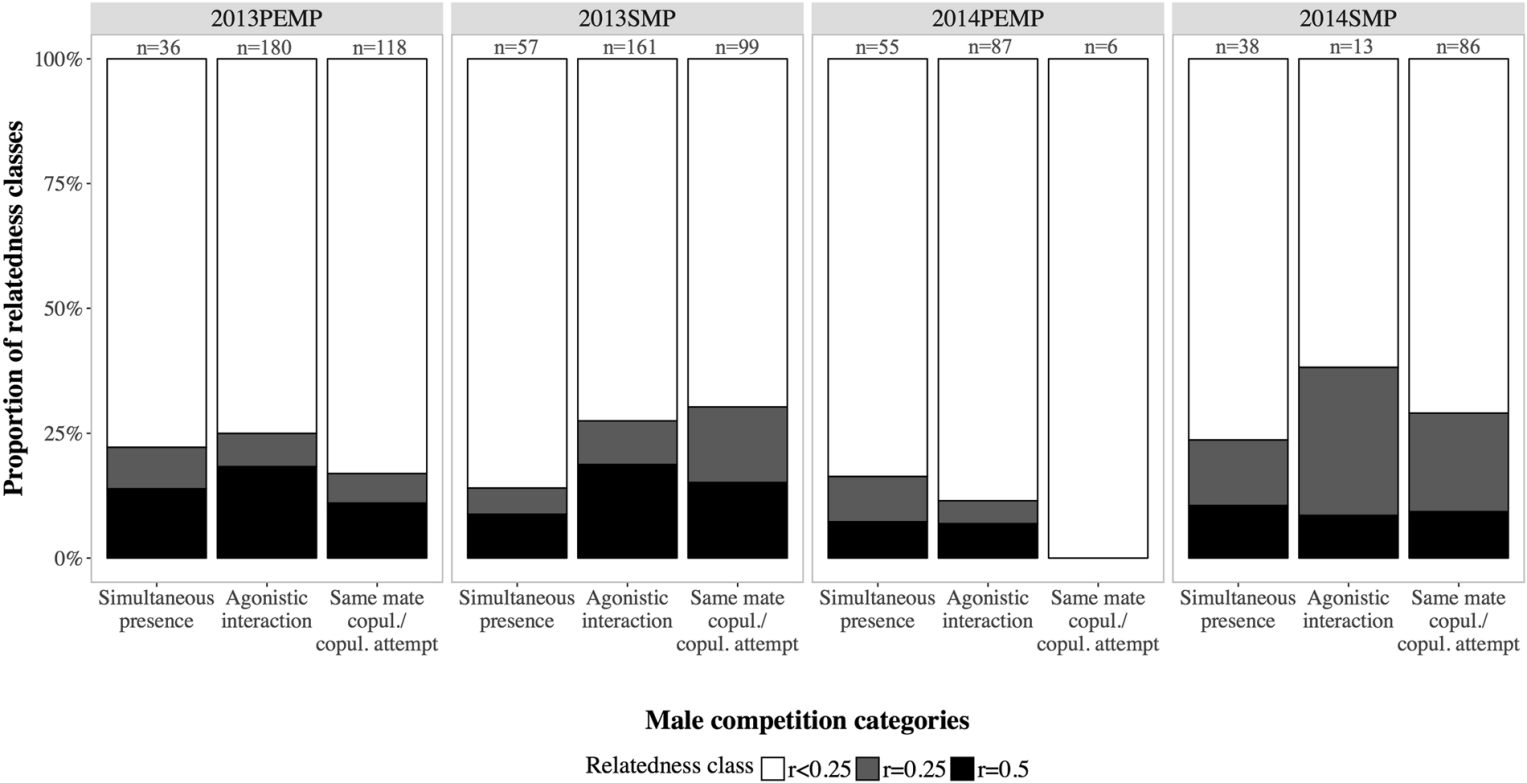
The proportion of relatedness classes of adult male dyads present within the same group and their direct competition. Both mating periods in 2013 and 2014 are shown [‘postpartum oestrous mating period’ (PEMP) and ‘seasonal mating period’ (SMP)]. The first bar (‘Simultaneous presence’) reflects the proportion of relatedness classes of male pairs that were simultaneously present in the same social group. The second bar (‘Agonistic interaction’) shows the proportion of relatedness classes of all agonistic interactions and the involved male pairs. The third bar (‘Same mate copulation/copulation attempt’) shows the proportion of relatedness classes of male pairs that copulated or attempted to copulate with the same female during the same mating period. The sample size of male pairs in each category and mating period is shown.

Our focus colony C5 consisted of two separate social groups defined by a constant set of bats with constant usage of the same sites within the roost (see Günther *et al*. 2016 for details on site usage). Since interaction and reproduction occurred virtually exclusively among members of each social group, we looked at mate competition and the relatedness class of the involved males within each social group separately. During four mating periods in 2013 and 2014, on average 80.3% (range: 73.7–86.0%) of all adult male pairs that were simultaneously present in the same social group were distantly related or unrelated (*r* < 0.25, see Fig. 6 for proportions of relatedness classes and sample sizes). Accordingly, on average 74.3% (range: 61.2–88.5%) of all dyadic agonistic interactions occurred between distantly related or unrelated male pairs (*r* < 0.25, see Fig. 6). Further, a median number of two different males (range: 1–4 males) copulated with the same female and a median of three different males (range: 1–6 males) attempted to copulate with the same female during the same mating period. However, on average 80.9% (range: 69.7–100%) of all male pairs that copulated or attempted to copulate with the same female were distantly related or unrelated (*r* < 0.25, see Fig. 6). Finally, during six mating periods between 2010 and 2014 a median number of three males reproduced simultaneously (i.e. sired at least one offspring during the same mating period, range: 1–6 males). Of all male pairs that reproduced simultaneously (*n* = 41), 85.4% were distantly related or unrelated, while 4.9% had a relatedness class of half-siblings (*r* = 0.25) and 9.8% were full-siblings or parent-offspring (*r* = 0.5).

To assess if males avoid mate competition with their male kin we tested whether pairwise relatedness had an impact on competition between males, we ran a ‘Double-Semi-Partialing Multiple Regression Quadratic Assignment Procedure’ (DSP-MRQAP^45^) for each mating period and each of the two groups in the focus colony C5. In each DSP-MRQAP we tested whether (1) the fact that two males share the same mother or (2) being father and son had an effect on (A) the ‘number of agonistic dyadic interactions’ or (B) ‘the fact that two males copulated or tried to copulate with the same female’. In both social groups, we did not find a significant effect of the three relatedness scenarios on competition scenario (A) or (B) (see supplementary Table S2 for details on results of all procedures).

## Discussion

Our long-term observations of individually marked bats in combination with genetic relatedness analyses reveal that on average 83.6% of males in *R. naso* colonies are philopatric. This leads to several lineages of closely related males per colony (i.e. patrilines, see Fig. 2) with simultaneous presence of several generations and even cases of simultaneous reproduction of closely related males. This represents a rare genetic and social structure in mammals^7^ and until now was shown mainly for highly social primate species^35^ but also in *R. naso*’s close relative, the greater sac-winged bat (*Saccopteryx bilineata* ^38^). Further, we show how male philopatry benefits colony born males on different levels, which in total leads to higher individual reproductive success of philopatric males compared to dispersing males.

Philopatric males are more successful regarding long-term colony integration compared to dispersing males. On average immigrant males disappeared already 76.7 days after arriving in the colony, while tenure of philopatric males was 2.07 years. This is an important advantage for philopatric males, because pups of a colony are usually fathered by resident males (between 2011 and 2014 there was only one case of an external male siring one offspring in our focus colony C5). Further, it illustrates possible costs for dispersing males through the hurdle of immigration into a foreign social group that can be averted by staying at the natal group. We speculate that the limited long-term integration probability could be the result of aggression by resident males against foreign intruders in which case all resident males would benefit from the aggression of other residents (i.e. by-product mutualism). Although mainly attributed to groups of kin^46^, many studies of birds and mammals show that cooperative behaviour also occurs between unrelated individuals (e.g.^47–53^, reviewed in^54,55^). In its simplest form this may occur as by-product mutualism, where for example males mutually benefit from keeping new competitors out of a social group without a cooperative investment (e.g.^55^). In *S. bilineata*, lower male immigration rates, longer tenure and higher lifetime reproductive success by harem males when more males are present in a colony suggests that harem males benefit from other male residents in excluding immigrant males from the colony^56^. Similarly, in the bat *Artibeus jamaicensis* satellite males less frequently approached harems defended by a dominant male that tolerated subordinate males in its harem territory^57^. As yet, we were not able to collect sufficient behavioural data on aggression towards recent immigrant males by resident males in *R. naso* roosts. Obtaining this data is difficult as (1) these incidents might be rare escalations during the first few days and likely to be missed, and (2) we expect aggression against immigrants mainly at night, when, however, behavioural observations are difficult. During the day, the high predation risk probably forces the bats to minimise conspicuous interactions (see^58,42^). *R. naso’s* morphological (camouflaged fur) and behavioural adaptations (remaining motionless unless there is a breeze of wind, during which bats stretch and groom in synchrony resembling shaking leafs) to remain visually cryptic^59,60^, suggest that predation pressure plays an important role during the day. Thus, the options for males in preventing other males from joining their group appear very restricted during the day, but are likely to be less restricted at night. Accordingly, recent immigrant males were present in the roost during day, but were never observed to be present in the roost at night. If recent immigrants are not tolerated in the social groups at night, this may explain their limited success in establishing residency. Thus, by-product mutualism among the resident males of a social group (kin and non-kin) may promote male philopatry in *R. naso*.

Long-term residency in a social group may be crucial for attaining a territory, as the average tenure until becoming territorial was 1.73 years. With a median tenure of only 76.7 days most immigrant males probably never get the chance to achieve this status. In contrast, with a median minimum tenure of 2.07 years, philopatric males have comparatively better chances. However, the probability of becoming a territorial male was not different between philopatric and immigrant males. In addition to assumingly increased chances of becoming territorial with increased tenure, a key factor in the males’ territorial mating strategy is the presence in the night roost and a constant occupation and defence of a specific site (territory) within the roost at night (i.e. high site fidelity, see^42^). While most immigrants were never observed to be present in the roost at night, philopatric non-territorial males were frequently present in the roost at night. This presence and familiarity with the night roost and the present bats might enhance the chances of taking over a vacant territory for philopatric *R. naso* males. Similarly, in the closely related *S. bilineata* where males queue to take over vacant harem territories it was also suggested that philopatric males benefit from familiarity with the nearby territories^61^.

Several empirical examples, reaching from simple mutual tolerance to complex cooperation in mating, breeding or foraging, support the notion that philopatric males may benefit from cooperating with their relatives (e.g. reviewed for nonhuman primates in^9^, red grouse, *Lagopus lagopus scoticus* ^10^). So far, we did not find any evidence for kin cooperation (e.g. mutual tolerance) in the context of mating behaviour in *R. naso*. None of the two kinship scenarios tested in the DSP-MRQAP analyses influenced agonistic interactions between males or whether males tried to copulate or copulated with the same female during the same mating period. This suggests, that *R. naso* males do not discriminate between kin and non-kin or exhibit any form of mutual tolerance towards closely related kin in the context of mating behaviour. Hence, kin cooperation is unlikely to be an ultimate factor for male philopatry in *R. naso* unless it occurs on a rather cryptic level and thus could not be observed.

Dispersing individuals have often been shown to be in worse physical conditions compared to philopatric males leading to lower fitness of dispersing males (reviewed in^13^). For instance, dispersing snowshoe hares (*Lepus americanus*) have less body weight, smaller adrenal glands and higher incidence of scarring^26^. In deermice (*Peromyscus maniculatus*) aggressive interactions between adults and juveniles delayed growth rates in juveniles^62^, which might affect the future success of these dispersing juveniles. Intriguingly, when measured as fully-grown adults, immigrant *R. naso* males were significantly smaller than philopatric males regarding forearm, third and fourth finger. For now, we can only speculate about the cause of this size difference. On the one hand, it may be the result of small males being competitively inferior and therefore displaying a greater dispersal propensity than larger and competitively superior males^27–29^. Correspondingly, Hanski *et al*.^27^ found that dispersers were smaller in skeletal traits and less competitive than resident common shrews (*Sorex araneus*). On the other hand, immigrant males may be smaller than philopatric males due to worse conditions during dispersal^13^. Regardless of whether the size difference is a cause or consequence of dispersal, smaller immigrant males in *R. naso* could be physically inferior and thus be less successful in long-term colony integration and competition for territories resulting in lower fitness compared to philopatric males.

Whereas a resource defence mating strategy prevails at night, *R. naso* males adopt a strategy of direct female defence during the day, with overt aggression between resident males and mate guarding^41,42^. Our study illustrates the presence of multiple reproductively active and agonistically interacting males with simultaneous copulations and copulation attempts in *R. naso* groups. Although one to two territorial males have the highest reproductive success^42^, up to six simultaneously reproducing males per mating period suggests that competition for access to females is severe. This and the fact that the majority of males in *R. naso* groups is philopatric with long tenures, raised the question of how related the competing males are and whether they thus face LMC. The low mean pairwise relatedness of male adults within the study colonies (similar values were found in group living apes with male philopatry^63,64^) suggests, that in spite of some closely related males (members of the same patriline) a substantial number of present males is distantly related or unrelated (members of different patrilines). In line with that, in our focus colony on average only 21.5% of the competing male pairs in the three competition categories ([1] ‘simultaneous presence’, [2] ‘agonistic interactions’, [3] ‘same mate copulations/copulation attempts’) were close kin with relatedness classes between *r* = 0.25 and *r* = 0.5. Furthermore, only 14.6% of simultaneously reproducing males had a relatedness classes between *r* = 0.25 and *r* = 0.5. Thus, although mate competition is high, owing to a high proportion of unrelated or distantly related males within the groups, the potential for LMC (i.e. competition for mates among kin) appears comparatively low. We suggest that the low average relatedness among the males of a social group is the result of multiple reproducing males with unrelated females (see^63^) and of rare, successful male immigration events. Also, *R. naso* males did not show any indication of avoiding competition with close kin. As tested in the DSP-MRQAP analyses, close kinship (i.e. father-son relationships or brothers sharing the same mother) had no effect on agonistic male-male interactions or the fact that males copulated or tried to copulate with the same female.

### Conclusion

The majority of males in *R. naso* colonies is philopatric and also reproduces therein leading to several patrilines with simultaneous presence of several closely related males. Philopatric males benefit from staying at their natal group through a significantly longer tenure and better integration (i.e. frequent nocturnal presence in the colonies) compared to immigrant males and consequently in significantly higher reproductive success. These differences might be caused by the hurdle of immigration into a foreign social group for dispersing males. This is likely to be the result of aggression by resident males towards foreign potential immigrant males causing by-product mutualism and the competitive disadvantages of dispersing males due to smaller body size. Thus, our results indicate proximate and ultimate advantages for philopatric males of staying at the natal group. Further, the lack of evidence for kin selected mutual tolerance in *R. naso* suggests that kin cooperation is unlikely to be an ultimate factor for male philopatry. Finally, our results show that in social groups comprised of unrelated females with multiple reproducing males and a small degree of male immigration, LMC – a supposedly costly factor for philopatric males with direct female defence as mating strategy – is comparatively low and may readily be outweighed by the benefits of staying at the natal group.

## Methods

### Field methods

The study was conducted between 2005 and 2016 in three main study colonies (Cabina 5, Riverstation, Casa Grande) and twelve additional colonies at the La Selva field station of the Organization for Tropical Studies (Costa Rica, Province Heredia, 10°25′N/84°00′W). Cabina 5 (C5) represents the focus colony of our study with the highest observation effort, the highest sampling and banding rate and almost complete offspring sampling for six mating periods. Thus, in the following method section the scope (i.e. the colonies) of data collection and analyses is mentioned in the beginning of each paragraph. Further, an overview with periods of different data collections and data sets used for specific analyses is provided as supplementary Figure S1 . In C5 and Riverstation (RS) the bats are located under the extending roof on the outside of an inhabited wooden station cabin and thus well habituated to human presence. Casa Grande (CG), the third study colony roosted in a partly destructed abandoned house on the wooden ceiling beams. These roost sites are known to be occupied by proboscis bats for at least 16 years. The 12 additional colonies are distributed over approximately 10 km along the banks of the Puerto Viejo River and the Sarapiqui River. Bats were mist netted (Ecotone® monofilament, Gdynia, Poland) in the vicinity of their roost, genetically sampled (Stiefel® biopsy punch, 4 mm Ø), individually marked with coloured plastic bands (AC Hughes® Ltd., UK, size XCS), sexed, measured (forearm, third and fifth finger) and their age class was determined (juvenile ≈ 0–4 months, subadult ≈ 5–10 months, or adult >10 months; see^41^ for details on all field procedures). Numbers of genetically sampled and banded bats between 2005 and 2014 are provided in Table 3.

**Table 3:** Number of banded and genetically sampled bats between 2005 and 2014.

Age	Sex	Banded	Sampled
Adult	Female	135	145
	Male	125	130
Subadult	Female	70	70
	Male	39	38
Juvenile	Female	55	65
	Male	57	71
Total		481	519

We did not band heavily pregnant females, juveniles of a too young age or individuals in bad body condition, thus more individuals have been sampled than banded. In four cases, we banded but failed to sample the bat.

### Census observation (C5, RS, CG)

We determined number of unbanded bats, number and identity of banded bats and motherhood of pups by nursing observations of banded mothers and pups on a daily to at least weekly basis between 2006 and 2016 during day (C5: *n* = 655 days, RS: *n* = 561 days; CG: *n* = 386 days; see supplementary Figure S1). A total of 185 night census observations were conducted in our focus study colony C5.

### Behavioural observations (Cabina 5)

During daytime observation sessions (on average 2–3 hours, evenly distributed across daytime) we recorded all visible behavioural interactions (ad libitum sampling sensu^65^) between bats of two social groups in the focus colony C5 in 2013 and 2014 (592:36 h in group 1, 532:46 h in group; see supplementary Figure S1). The two groups were defined based on the observation of constant usage of the same sites within the roost by the same set of bats over each observation period (see^42^ for details). It was possible to observe all group members at the same time since all group members clustered within small assessable areas (approx. 1–3 m^2^). All physical interactions were fairly easy to observe, owing to *R. naso’s* spacing behaviour (5–10 cm individual distance). Since the groups split up between different sites at night, night observations were focused on specific sites within the roost during 20 nights in September-November 2014 (23:02 h). In addition, the whole roost was constantly checked for behavioural interactions during 12 nights in October and November 2014 (24:32). See Günther *et al*.^42^ for details on methods of behavioural observations.

### Census analyses

#### Age of disappearance (Cabina 5)

We determined the duration from birth until disappearance (i.e. the last day of observation in the natal colony without returning until the end of the study in November 2016) from the natal colony for 15 females and 29 males. In subsequent analyses, we use this as a proxy for dispersal age allowing us to define philopatric individuals, but as we rarely knew whether an individual died or dispersed we use the term disappearance. We included all individuals that were observed to be born in the colony and subsequently banded (*n* = 15 females, *n* = 27 males) or determined by genetic assignment of the mother in the colony (*n* = 2 males).

#### Minimum male tenure (C5, RS, CG)

We determined the minimum tenure as the time interval between the day of banding and the last day of observation of the bat in its colony for all males caught between 2005 and 2014 for which we had information on dispersal status (i.e. philopatric males: *n* = 47 and immigrant males: *n* = 17). We use this as a proxy for the success of males regarding long-term integration into a colony. The census observations for male tenure duration ended in October 2015 (RS), July 2016 (CG) and November 2016 (C5). Males that were either observed to be born and/or nursed in their current colony and that stayed longer than the maximum age of female dispersal (*n* = 16) or males caught without nursing observation but genetic determination of the mother in their current colony (*n* = 31) were considered philopatric. Males that were either observed immigrating into the colony (*n* = 13) or caught in the colony and assigned genetically to at least one parent in another colony (*n* = 2) or no mother was assigned although all mothers were sampled during the mating period of conception (*n* = 2) were considered immigrants. For the comparison of tenure estimates among philopatric and immigrant males, we used the day of average dispersal age of females as beginning of tenure for philopatric males (*n* = 17). Bats that disappeared before the end of the field season of their banding were not included in tenure calculations (*n* = 8 philopatric males, *n* = 2 immigrant males), because their disappearance was probably caused by the disturbance of being captured. Nevertheless, short-term immigrants that stayed only several days or weeks and disappeared without any disturbance are included in the tenure calculations as we attempted to also document failed immigration events.

#### Minimum tenure until territoriality/as territorial male (Cabina 5)

We calculated median minimum tenure until becoming territorial for four males with information on their start of being territorial between 2013 and 2016. Territoriality was determined by the high fidelity of a male towards a single site in the roost at night. In contrast, non-territorial males were either absent at night or switched among sites and used two to four different sites in the roost at night (see Günther *et al*.^42^ for more details on determination of territorial males). Four additional males were already territorial in 2013. We calculated median minimum tenure as a territorial male for a total of eight territorial males between 2013 and 2016.

### Behavioural analyses (Cabina 5)

We differentiated between: (1) Copulations, (2) copulation attempts rejected by females and (3) agonistic male-male interaction. These behavioural events were counted individually while start and endpoints were recognised as subsequently described. Copulation: A male approaches a female from behind, subsequently mounts her back, flattens its interfemoral membrane, gives several short thrusts and finally taps the female with his chin on her back and retracts from her voluntarily. Copulation attempt: A male approaches and mounts the female’s back but is rejected by the female by hitting with a wing and/or the female’s escape (flying/crawling). Agonistic male-male interactions: One male quickly approaches another male by directly crawling or flying towards the male, either resulting in a physical contact with hitting and subsequent separation of the two males or the attacked male avoids physical contact by flying/crawling away. The speed and directedness of the approach allowed us to distinguish an attack from a simple relocation of a bat in the roost.

### Kinship analyses (C5, RS, CG)

For the following parentage analyses we used microsatellite data of 519 individuals genotyped at ten highly polymorphic microsatellite loci (mean = 24.4 alleles; range: 11–64 alleles/locus) published by the authors in a prior study^66^. We used CERVUS v. 3.0^67^ – a simulation based maximum likelihood approach – for parentage analyses of 139 bats caught between May 2011 and November 2014 as juveniles (*n* = 89) and subadults (*n* = 50). For maternity analyses all ten loci were used, while one x–linked gonosomal locus (Rn16) was left out for male–male paternity assignments^68^. To achieve highest reliability of paternity assignments, we first determined maternity and used this information for the subsequent determination of paternity. Adding to the confidence of the parentage analyses, nursing observations of 62 juveniles and their mothers were all confirmed by the genetic analyses. Maternity was analysed without prior nursing observations for 27 juveniles and 50 subadults. For the subsequent paternity assignment of juveniles and subadults with known mother (*n* = 90) and with unknown mothers (*n* = 49), all males that were adult during the respective mating period when potential colony offspring were conceived were treated as putative fathers (*n* = 162). Simulations with 100 000 cycles were performed with a proportion of 80% sampled candidate fathers (to account for possible extra-colony paternities), a genotyping error of 2.2% (estimated with CERVUS v. 3.0 based on 22 mismatches between 80 known mother offspring pairs), and a proportion of 14.5% candidate fathers that were relatives, related to the true father by *r* = 0.5 (estimate from^41^). Simulations were performed for two confidence levels (80% and 95%) to assess the degree of uncertainty in the assignments. Only one mismatch per parent–offspring dyad was accepted, thus two independent mismatches between an offspring and its parents to account for genotyping errors. Seventy-nine parent pairs were assigned at 95% confidence, and three parent pairs were assigned at 80% confidence. For two subadult and five juvenile individuals only the mother was assigned at 95% confidence, and for seven juvenile individuals only the father was assigned at 95% confidence. All these parent-offspring assignments were positively checked for plausibility (i.e. presence of the assigned parents in the regarding mating period of conception).

In order to reconstruct patrilines, parentage analyses were also performed for all males caught as adults between 2005 and 2014 (*n* = 126). This resulted in 23 adult males with assigned parent pairs at 95% confidence and four father-son relationships at 95% confidence without an assigned mother. For 99 of all males sampled as adults we found neither mother nor father. The patriline illustrations (see Fig. 2) reflect the period between 2011 and 2014 (RS and CG) and 2011 and 2015 (C5), respectively. This period was chosen owing to consistently high sampling and census rates of the study colonies starting from 2011 (mean: 87.7%, see supplementary Figure S1). Still, adult male members of patrilines present before these periods are included. This is important to assess the number of patrilines and relatedness of present males as realistic as possible. Based on the patriline reconstruction and parentage data derived from CERVUS, we determined all relatedness classes (*r* = 0.5, 0.25 and 0.125) beside parent-offspring pairs.

In addition to the relatedness classes derived from patriline reconstructions we used KINGROUP v2^69^ to estimate pairwise relatedness between males. KINGROUP is based on the likelihood-calculation algorithm of the software KINSHIP^70^. Pairwise relatedness was estimated as continuous *r*-value sensu Wang^71^ and used to calculate the mean pairwise relatedness between adult males present in the same colony during the same mating period. For this calculation, well-sampled mating periods with census observations were used (in C5, RS, CG: PEMP 2011, PEMP 2013, SMP 2013, PEMP 2014, SMP 2014; only in C5: SMP 2015; see also supplementary Figure S1).

### Multiple regression quadratic assignment procedure – MRQAP (Cabina 5)

To test whether kinship had a significant effect on male-male competition we used a ‘Double-Semi-Partialing Multiple Regression Quadratic Assignment Procedure’ (DSP-MRQAP^45^). This procedure is similar to the Mantel test but due to the potential of autocorrelation of relational dyadic data the assumption of independence can be violated and p-value estimates biased. Thus, the DSP-MRQAP uses a Monte Carlo method, which randomly permutes rows and columns within matrices, to determine the significance of regression coefficients (Dekker *et al*.^45^). For the analyses with 1,000 permutations we tested the effect of two different kinship scenarios on two different competition scenarios. ‘Kinship scenario 1’ reflects father-son relationships (see supplementary Table S3). To account for the possibility that males recognise their mother and two males might recognise their brotherhood through their mother, ‘kinship scenario 2′ is based on males who share the same mother (see supplementary Table S3). ‘Competition scenario A’ is based on the number of agonistic dyadic interactions (see supplementary Table S4–S11) and reflects the extent of aggression between male dyads. ‘Competition scenario B’ is based on the fact that two males copulated or tried to copulate with the same female during the same mating period (see supplementary Table S12–S19). Using the ‘asnipe’ package of R^72,73^, each kinship scenario was regressed against each competition scenario separately in univariate models and against the competition scenarios in combination with multivariate analyses (see supplementary Table S2). Each test was performed for the two social groups in C5 and the four mating periods in 2013 and 2014 separately.

### Other statistical analyses

All other statistical analyses were performed with *R* ^©^ version 3.3.2^73^. Data distribution was tested with the Shapiro–Wilk normality test. Mann–Whitney *U*–tests with continuity correction were used to examine the median difference of non–normally distributed data. Medians are presented either with minimum and maximum values or interquartile range (IQR) in the form of first quartile to third quartile (i.e. IQR = Q1–Q3). Whiskers in boxplots show Q_1_ minus 1.5 times IQR or Q_3_ plus 1.5 times IQR, respectively. Outliers are defined as values beneath or above the ‘1.5 cut–off’.

### Data availability

Microsatellite data of typed individuals (ID, sex, sampling location and alleles of 10 loci) is deposited at Dryad Digital Repository: http://dx.doi.org/10.5061/dryad.df878.

### Ethical standards

All field work was approved by the Costa Rican authorities (MINAET Ministerio del Ambiente, Energia y Telecomunicaciones and SINAC Sistema Nacional de Areas de Conservación, Permit number: 022–2005-OFAU, 108–2006-SINAC, 147–2007-SINAC, 183–2008-SINAC, 187–2009-SINAC, 130–2010-SINAC, 068–2011-SINAC, 115–2012-SINAC, 033–2013-SINAC, SINAC-SE-GASP-PI-R-121–2013, R-006–2015-OT-CONAGEBIO, SINAC-SE-CUS-PI-R-088–2016) and was in compliance with the current laws of Costa Rica and Germany.

## Electronic supplementary material

Supplementary Information
