# Supplementary material for: The evolution of a rare mammalian trait – benefits and costs of male philopatry in proboscis bats
Source: Sci Rep. 2017 Nov 15;7:15632. doi: 10.1038/s41598-017-15990-6 (PMC5688083; doi:10.1038/s41598-017-15990-6)
Supplement: Supplementary file 1 — Supplementary Information [file 41598_2017_15990_MOESM1_ESM.pdf]

Supplementary information

The evolution of a rare mammalian trait – benefits and costs of male philopatry in proboscis bats

Linus Günther*, Mirjam Knörnschild, Martina Nagy, Frieder Mayer

Figure S1. An overview of the type of data that was collected, the sampling rate and the effort that was made, the periods that were covered and the periods that were used for the different analyses. The scope regarding the covered study colonies is shown [Cabina 5 (C5), Riverstation (RS), Casa Grande (CG) and the twelve additional colonies (12 add. colonies)]. Cabina 5 is separated into two social groups based on behavioural observations during the day. For analyses in the context of mating behaviour the used mating periods are given [‘postpartum oestrous mating period’ (PEMP) and ‘seasonal mating period’ (SMP)].

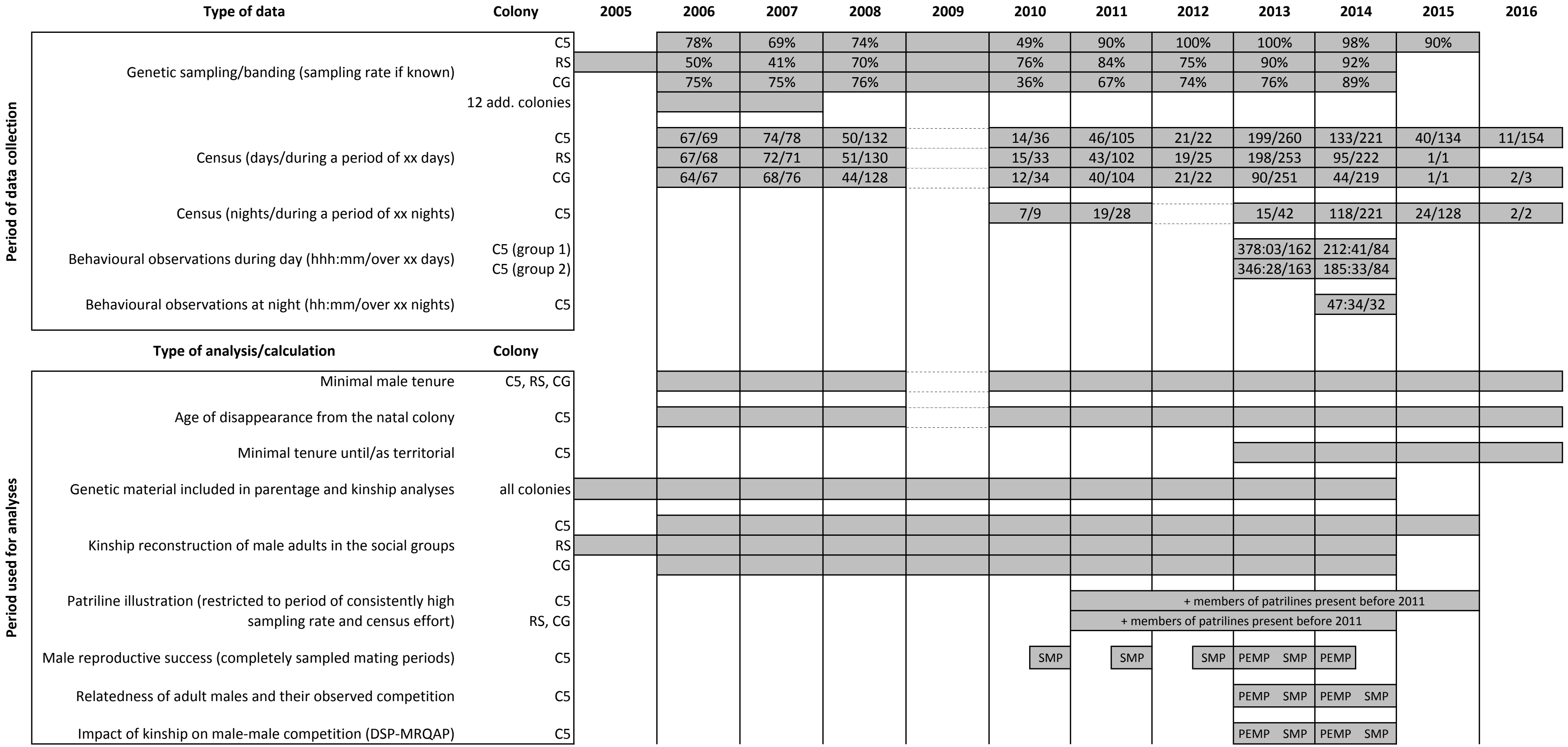

Table S2. The effect of kinship scenario 1 (father-son relationship) and scenario 2 (males sharing the same mother) on competition scenario A (number of agonistic dyadic male-male interactions) and competition scenario B (the fact that two males copulated or tried to copulate with the same female during the same mating period). Regression coefficients and p-values were estimated based on MRQAP-DSP permutation tests (Dekker *et al.* 2007). Tests were performed separately for the two social groups in the focus colony C5 and the four mating periods between 2013 and 2014. Columns 3-6 show the effects of each kinship scenario on the two competition scenarios tested separately in univariate models. Columns 7-10 show the effects tested together in a multivariate model including all kinship scenarios. Due to an insufficient number of pairs sharing any kinship or competition scenario (i.e. less than 1-4 pairs depending on the covariance between the matrices), it was not possible to calculate all models in some periods (see 'N/A'). Additionally, due to insufficient observations of copulations/copulation attempts in group 1 during PEMP 2014 (see table S14), we did not calculate the models in this case (see 'N/D').

		Univariate Model (Kinship Scenario 1+2 separately)				Multivariate Model (Kinship Scenario 1+2)			
		Kinship Scenario 1		Kinship Scenario 2		Kinship Scenario 1		Kinship Scenario 2	
Competition Scenario A (agonistic male-male-interaction)	Period	Coefficient	*p*-Value	Coefficient	*p*-Value	Coefficient	*p*-Value	Coefficient	*p*-Value
Group 1	PEMP 2013	-0.43	0.45	-0.25	0.46	0.14	1.00	N/A	N/A
	SMP 2013	0.58	0.12	-0.13	0.59	-1.76	0.96	-1.25	0.71
	PEMP 2014	0.07	0.67	0.27	0.30	-0.50	0.29	-0.01	0.98
	SMP 2014	0.17	0.65	-0.19	0.55	-0.14	0.97	-0.32	0.62
Group 2	PEMP 2013	-0.23	0.61	0.86	0.12	-1.00	0.81	N/A	N/A
	SMP 2013	-0.32	0.26	-0.30	0.57	-1.60E-16	1.00	N/A	N/A
	PEMP 2014	N/A	N/A	-0.11	N/A	N/A	N/A	N/A	N/A
	SMP 2014	N/A	N/A	-0.13	0.73	N/A	N/A	-0.50	0.55
Competition Scenario B (same mate copulation/cop. attempt)									
Group 1	PEMP 2013	-0.24	0.54	0.17	0.52	-0.50	1.00	N/A	N/A
	SMP 2013	-0.08	0.94	0.29	0.27	1.33	0.98	0.97	0.92
	PEMP 2014	N/D	N/D	N/D	N/D	N/D	N/D	N/D	N/D
	SMP 2014	-0.18	0.62	0.08	0.91	0.56	0.72	0.44	0.51
Group 2	PEMP 2013	-6.62E-17	1.00	-6.14E-17	1.00	1.36E-31	0.981	N/A	N/A
	SMP 2013	0.21	0.41	0.40	0.08	-0.2	0.986	N/A	N/A
	PEMP 2014	N/A	N/A	-0.11	N/A	N/A	N/A	N/A	N/A
	SMP 2014	N/A	N/A	0.00	1.00	N/A	N/A	0.00	1.00

Table S3. Kinship scenarios [father-son pairs (PO) and brothers sharing the same mother (Bros)]. Father-son pairs are used as 0,1-matrix for 'kinship scenario 1'. Two males sharing the same mother are used as 0,1-matrix for 'kinship scenario 2'. Males that were present as adults during at least one of the four mating periods between 2013 and 2014 in the two social groups at the focus colony C5 are shown.

ID	217	338	389	373	415	453	444	210	305	342	484	306	371	404	421	418	472	332	445	370	382	396	394	440	315	455	446	495	473	462	505
217		PO	PO	PO		PO	PO																								
338		-		Bros	Bros																							PO			
389			-																												
373				-	Bros																										
415					-																										
453						-	Bros																								
444							-																					Bros			
210								-	PO																						
305									-	Bros				Bros					Bros								PO				
342										-	PO			Bros					Bros							PO					
484											-																				
306												-	PO			PO	PO												PO		
371													-	PO	PO		Bros												Bros		
404														-					Bros												
421															-											Bros					
418																-															
472																	-												Bros		
332																		-	PO												
445																			-												
370																				-											
382																					-										
396																						-									
394																							-								
440																								-							
315																									-						
455																										-					
446																											-				
495																												-			
473																													-		
462																														-	
505																															-

Tables S4-S11. Competition scenario A. The number of agonistic dyadic male-male interactions in the two social groups of the focus colony C5 during both mating periods [‘postpartum oestrous mating period’ (PEMP) and ‘seasonal mating period’ (SMP)] in 2013 and 2014.

S4 Group 1 – PEMP 2013

ID	305	210	332	342	371	404	389
305	-	0	5	0	10	11	14
210		-	6	0	2	2	0
332			-	0	2	1	0
342				-	0	0	3
371					-	6	2
404						-	1
389							-

S5 Group 1 – SMP 2013

ID	305	210	332	342	394	371	440	404	389
305	-	4	1	6	0	6	3	8	11
210		-	14	4	2	1	4	4	2
332			-	1	4	0	4	2	8
342			1	-	3	1	1	1	10
394					-	2	1	1	1
371						-	0	2	5
440							-	2	0
404								-	11
389									-

S6 Group 1 – PEMP 2014

ID	421	305	210	332	342	415	371	404	418	389
421	-	0	4	1	0	1	0	0	0	0
305		-	2	5	0	2	1	2	1	2
210			-	4	1	5	0	1	2	1
332				-	1	0	0	1	1	4
342					-	1	0	2	1	1
415						-	1	0	0	0
371							-	2	0	0
404								-	0	6
418									-	0
389										-

S7 Group 1 – SMP 2014

ID	421	455	210	342	415	371	445	505	370
421	-	0	0	3	4	4	1	4	1
455		-	0	0	0	0	0	0	0
210			-	1	10	1	2	0	0
342				-	8	0	1	0	1
415					-	5	14	1	3
371						-	1	0	1
445							-	0	0
505								-	0
370									-

S8 Group 2 – PEMP 2013

ID	338	306	217	373	370	382
338	-	4	5	2	2	4
306		-	17	4	3	9
217			-	18	7	19
373				-	1	9
370					-	2
382						-

S9 Group 2 – SMP 2013

ID	338	306	396	217	418	373	370	382
338	-	6	0	17	7	0	2	5
306		-	5	6	4	12	4	12
396			-	0	0	1	1	0
217				-	2	7	4	3
418					-	1	3	0
373						-	7	1
370							-	4
382								-

S10 Group 2 – PEMP 2014

ID	338	373	315	370	382
338	-	4	3	3	1
373		-	0	0	3
315			-	0	1
370				-	0
382					-

S11 Group 2 – SMP 2014

ID	453	338	444	373	382
453	-	15	7	6	13
338		-	15	5	7
444			-	2	4
373				-	5
382					-

Tables S12-S19. Competition scenario B. Males that tried to copulate or copulated with the same female in the two social groups of the focus colony C5 during four mating periods between 2013 and 2014. A ‘1’ indicates that both males tried to copulate or copulated with an identical female at least once.

S12 Group 1 – PEMP 2013

ID	305	210	332	342	371	404	389
305	-	0	1	1	1	1	1
210		-	0	0	0	0	0
332			-	1	1	0	1
342				-	1	0	1
371					-	1	1
404						-	1
389							-

S13 Group 1 – SMP 2013

ID	305	210	332	342	394	371	440	404	389
305	-	1	1	1	0	0	1	1	1
210		-	1	1	0	0	0	1	1
332			-	1	0	0	1	1	1
342				-	0	0	1	1	1
394					-	0	0	0	0
371						-	0	0	0
440							-	0	1
404								-	1
389									-

S14 Group 1 – PEMP 2014

ID	421	305	210	332	342	415	371	404	418	389
421	-	0	1	0	0	0	0	0	0	0
305		-	0	0	0	0	0	0	0	0
210			-	0	0	0	0	0	0	0
332				-	0	0	0	0	0	0
342					-	0	0	0	0	0
415						-	0	0	0	0
371							-	0	0	0
404								-	1	0
418									-	0
389										-

S15 Group 1 – SMP 2014

ID	421	455	210	342	415	371	445	505	370
421	-	0	0	0	0	0	0	0	0
455		-	0	0	0	0	0	0	0
210			-	1	1	1	0	0	1
342				-	1	1	0	0	1
415					-	1	1	0	1
371						-	1	0	0
445							-	0	0
505								-	0
370									-

S16 Group 2 – PEMP 2013

ID	338	306	217	373	370	382
338	-	1	1	1	1	1
306		-	1	1	1	1
217			-	1	1	1
373				-	1	1
370					-	1
382						-

S17 Group 2 – SMP 2013

ID	338	306	396	217	418	373	370	382
338	-	1	0	1	0	1	0	1
306		-	0	1	0	1	0	1
396			-	0	0	0	0	0
217				-	0	1	0	1
418					-	0	0	0
373						-	0	1
370							-	0
382								-

S18 Group 2 – PEMP 2014

ID	338	373	315	370	382
338	-	0	0	1	1
373		-	0	0	0
315			-	0	0
370				-	1
382					-

S19 Group 2 – SMP 2014

ID	453	338	444	373	382
453	-	1	1	1	1
338		-	1	1	1
444			-	1	1
373				-	1
382					-
